# Family history of venous thromboembolism and mortality after venous thromboembolism: a Swedish population-based cohort study

**DOI:** 10.1007/s11239-016-1464-y

**Published:** 2016-12-19

**Authors:** Bengt Zöller, Mirnabi Pirouzifard, Jan Sundquist, Kristina Sundquist

**Affiliations:** Center for Primary Health Care Research, Skåne University Hospital, Lund University/Region Skåne, CRC, Building 28, Floor 11, Entrance 72, 205 02 Malmö, Sweden

**Keywords:** Venous thromboembolism, Deep venous thrombosis, Pulmonary embolism, Mortality, Family history, Genetics, Comorbidity

## Abstract

Studies on whether family history (FH) of venous thromboembolism (VTE) affects long-term mortality after VTE are missing. The aim of this study was to determine whether FH of VTE affects long-term mortality after a first episode of VTE. Using Swedish medical databases, we conducted a 30-year nationwide cohort study of 49,159 adult Swedish born patients included in the multi-generation register (born 1932 or later) with a first-time VTE (1981–2010). Using Cox regression, we assessed mortality Hazard ratios (HRs) with 95% confidence intervals (CIs). Totally 10,093 (20.5%) patients with VTE had a first-degree FH of VTE (parent/sibling). Patients without FH of VTE had significantly more VTE provoking risk factors and comorbidities than those with FH. The mortality HR the first 10-years after first time VTE was decreased for those with FH of VTE compared to for those without FH: crude HR 0.807, 95% CI 0.771–0.845 and adjusted HR 0.864, 95% CI 0.826–0.905. After 10-years of follow-up there was no significant effect of FH of VTE on mortality: crude HR = 1.018, 95% CI 0.905–1.145 and adjusted HR = 0.995, 95% CI 0.884–1.119. Cancer-associated mortality was more common in those without FH the first 10 years (56.9 vs. 53.4%, p = 0.002). After 10 years there were no difference in cancer-associated mortality (4.9 vs. 5.6%, p = 0.604). The results suggest that patients with FH of VTE have lower thrombotic threshold and need less provoking factors and comorbidities. They have also slightly lower total and cancer mortality the first 10 years after VTE.

**Electronic supplementary material:**

The online version of this article (doi:10.1007/s11239-016-1464-y) contains supplementary material, which is available to authorized users.

## Introduction

Venous thromboembolism (VTE), including deep venous thrombosis (DVT) and pulmonary embolism (PE), is a frequent occurring cardiovascular disease [1]. It is a multicausal disease caused by a number of interacting acquired and inherited risk factors [1]. A number of studies have reported the long-term mortality after VTE [2–14]. However, no study has reported whether family history of VTE affects the mortality and prognosis of a first time VTE. Several studies have shown that family history of VTE is a risk factor for first time VTE with a two- three-fold increased risk for primary VTE when a first-degree relative is affected, reviewed by Zöller et al. [15]. Though family history of VTE is linked to major or classical thrombophilias it is an independent risk factor for VTE [15–17]. Family history of VTE is a less important risk factor for recurrent VTE [18–22]. Two studies have found no such association while three studies have found a slight to moderate effect only [18–22].

We undertook this nationwide population-based cohort study to examine 30-year VTE mortality according to family history of VTE when controlling for VTE subtypes, underlying provoking factors and comorbidities.

## Methods

We used data from several Swedish registries linked by the unique individual Swedish personal identity number assigned to all residents of Sweden (at birth or immigration) [23–27]. A random number replaced the personal identity number in order to preserve confidentiality. Linking the following sources created the database used in the present study: the Total Population Register; the Multi-Generation Register; the Swedish Hospital Discharge Register; and the Swedish Register of Causes of Death. The Swedish hospital discharge records contains diagnosis at discharge classified according to the International Classification of Diseases (ICD), 8th revision (1969–1986), 9th revision (1987–1996) and 10th revision (1997–2010). The Swedish Total Population Register provided information about sex, country of birth, mortality and birth year.

### Inclusion of patients

Patients were born 1932 or later. Only cases with a first time VTE were included at age of 18 years or older. VTE was defined as a main or secondary diagnosis of Deep Venous Thrombosis (DVT), Pulmonary Embolism (PE), or a combination of DVT and PE (PD) in the hospital discharge register occurring 1981–2010. Deep Venous Thrombosis (DVT) was defined as the following (ICD-8: 451), (ICD-9: 451) and (ICD-10: I80). Pulmonary Embolism (PE) was defined as the following (ICD-8: 450), (ICD-9: 415B) and (ICD-10: I26). Combination of DVT and PE (PD) was defined as the following (ICD-8: 450, 451), (ICD-9: 415B, 451) and (ICD-10: I26, I80). Patients with only superficial thrombosis (ICD-10 code I800 or ICD-9 code 451A) were excluded. Patients with a VTE between 1968 and 1981 were excluded. Only Swedish born individuals were included. No information about ethnicity exist in the Swedish registers, only country of birth. Adopted patients were also excluded. A total 245,156 individuals with first time VTE diagnosis at age 18 years or older between 1981 and 2010 were identified from the Hospital Discharge Register. Of those individuals we identified 49,159 patients (offspring) who meet the inclusion criteria as named above. The date for first time VTE we call the index date.

### Ascertainment of cases

The validity of the Swedish Hospital Discharge register is high and especially for cardiovascular disorders including VTE the validity is around 90–95% [25, 27, 28].

### Main predictor

The Swedish Multigeneration Register, which contains information on family relationships, was used to assess the main predictor. The register contains information on index persons registered in Sweden after 1 January 1961 and born on or after 1 January 1932 [24]. Family history of VTE was defined as a main or secondary diagnosis of VTE (DVT or PE) in the hospital discharge register occurring any time between 1968 and 2010 in adult first-degree relatives (siblings and/or parents) at age of 18 years or older by ICD codes for VTE described above. Main and secondary diagnosis in the hospital discharge register were included.

### Co-variates included in the analysis

Included co-variates were age, sex, education, type of first time VTE manifestation (DVT, PE, or PD, see above), and comorbidities. Comorbidities were defined by main or secondary diagnosis in the Swedish Hospital discharge register according to ICD-8, ICD-9 or ICD-10 codes occurring between 1968 and first event of VTE (i.e. at index date). The ICD codes for the included comorbidities are listed in Supplement Table 1. The studied comorbidities were cancer, hypertension, atrial fibrillation/flutter, obesity, psychiatric disease, coronary heart disease (CHD), heart failure, psychiatric disease, peripheral vascular disease, cerebrovascular disease, liver disease, varicose veins, inflammatory bowel disease (IBD), asthma, and other pulmonary diseases (chronic obstructive pulmonary disease, emphysema, bronchiectasis, interstitial pulmonary disease, and pneumoconiosis). Pregnancy/delivery, fractures/trauma, and surgery needed to occur within 90 days before first time VTE (the index date).

**Fig. 1:**
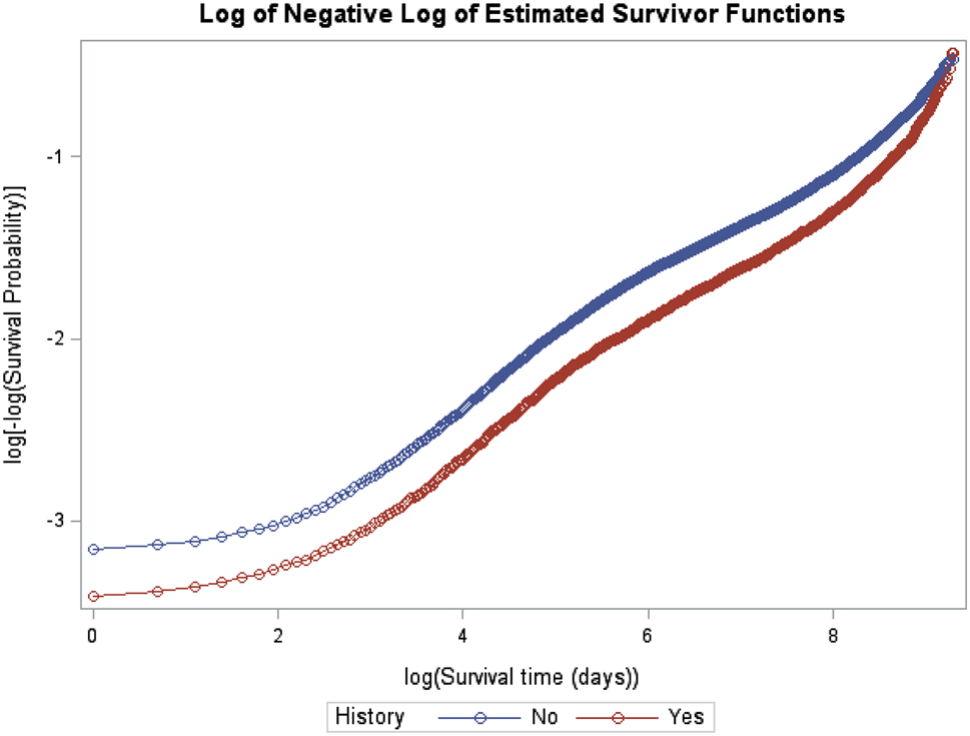
Logarithm (Log) of negative logarithm of estimated survivor functions. This Log–Log plot indicates that the *curves* diverge after 3650 days (10 years)

**Table 1:** Characteristics of patients (N = 49 159) with first time venous thromboembolism (VTE), i.e. deep venous thrombosis (DVT), pulmonary embolism (PE), or combined DVT and PE (PD)

	No % (39,066)	Yes % (10,093)	P
History (49,159)			
Diagnosis			
DVT	46.6	46.6	<0.001
PD	5.1	6.2	
PE	48.4	47.3	
Sex			
Female	49.0	46.4	<0.001
Male	51.0	53.6	
Age			
Median (IQR)	55 (44–63)	55 (46–63)	
Education			
Low (0–9 years)	32.0	34.2	<0.001
Middle (10–11 years)	44.6	44.1	
Higher (12 years or more)	22.9	21.2	
Unknown	0.6	0.5	
Asthma^a^	3.5	3.0	0.018
Atrial fibrillation^a^	4.9	4.4	0.030
Cancer^c^	20.8	17.3	<0.001
Cerebrovascular disease^a^	6.2	6.5	0.333
Congestive heart failure^a^	4.1	3.5	0.005
Coronary Heart disease^a^	7.6	6.8	0.005
Diabetes^a^	7.2	6.5	0.012
Fractures/Trauma^b^	6.7	5.9	0.005
Hypertension^a^	14.4	14.4	0.917
Inflammatory bowel disease^a^	2.3	2.0	0.068
Liver disease^a^	1.2	0.9	0.014
Obesity^a^	2.1	2.0	0.670
Peripheral vascular disease^a^	3.6	3.8	0.351
Pregnancy/delivery^b^	1.4	1.1	0.059
Psychiatric disease^a^	14.0	13.2	0.036
Pulmonary disease except asthma^a^	4.0	3.9	0.576
Varicose veins^a^	2.8	3.2	0.033
Surgery^b^	16.9	15	<0.001

^a^Any time before or at index date

^b^Within 90 days before and at index date

^c^5 years before or at index date

### Statistical analysis

We used Cox proportional-hazards models to calculate hazard ratios (HRs) and 95% confidence intervals [29]. The Cox model time scale was follow-up time from the first diagnosis registration between 1981 and 2010, until emigration, death, or the end of follow-up (December 31, 2010), whichever occurred first. In all models, we investigated the proportional hazards assumption by including an interaction term between diagnosis and the time. However, there was a significant interaction between time and family history of VTE (p < 0.001). The log of negative log of survival plot without adjusting for other covariates in Fig. 1 show that the survival curves seems to diverge after 3650 days (10 years). The graphical approach indicates that the assumption of proportionality over time is violated. Thus, time affects the relative mortality risk and we can conclude that the risks in our model are not proportional. We therefor considered time-dependent variables for diagnosis and used an extended cox model (Heaviside functions). Multivariate models were adjusted for age, sex, education, type of first VTE (DVT; PE; PD), comorbidities and provoking VTE risk factors. SAS, version 9.3 (SAS Institute, Inc., Cary, North Carolina) was used for analyses.

### Ethical approval

The study was approved by the Ethics Committee of Lund University, Sweden (approval number 409/2008, with amendments approved on September 1 2009 and January 22 2010). It was performed in compliance with the Declaration of Helsinki. Consent was not obtained but the presented data were anonymized thus eliminating any risk of identification.

## Results

### Patient characteristics

Between years 1981 and 2010, a total of 49 159 VTE patients (offspring), born in Sweden and aged 18 years or older, were identified on the basis of their first discharge recorded in the Hospital Discharge Register (Table 1). Among the identified VTE patients, 39 066 (79.5%) patients included had no family history of VTE (NH) and 10 093 (20.5%) VTE patients had a family history (YH) of VTE. There were slightly more females (49.0%) in NH group than in the YH group (46.4%). Median age among NH patients was 55 years with Interquartile Range (IQR) (44–63 years) and 55 years (IQR 46–63 years) among YH patients. A higher proportion of patients with family history of VTE had PD than patients without family history (6.2 vs. 5.1%). A similar proportion of patients had manifestations of pulmonary embolism (PE + PD: 53.5 vs 53.5%) but a higher proportion had DVT among patients with family history of VTE (DVT + PD: 52.8 vs. 51.7%). There were also a slightly higher proportion of individuals with low education among those with family history of VTE.

### Comorbidities and provoking risk factors

Several comorbidities or provoking risk factors were slightly but significantly more common among patients with no family history of VTE than among those with family history: asthma (3.5 vs. 3.0%), atrial fibrillation/flutter (4.8 vs. 4.3), cancer (20.8 vs. 17.3), congestive heart failure (4.1 vs. 3.5) coronary heart disease (7.6 vs. 6.8%), diabetes mellitus (7.2 vs. 6.5%), fracture/trauma (6.7 vs. 5.9%), pregnancy/delivery (1.4 vs. 1.1%), psychiatric disease (14.0 vs. 13.2), liver disease (1.2 vs 0.9%), and surgery (16.9 vs. 15%). The opposite relationship was observed for varicose veins. Patients in YH group had slightly but significantly more varicose varices (3.2% vs. 2.8%) than patients in NH group.

### Mortality after a first event of VTE

**Fig. 2:**
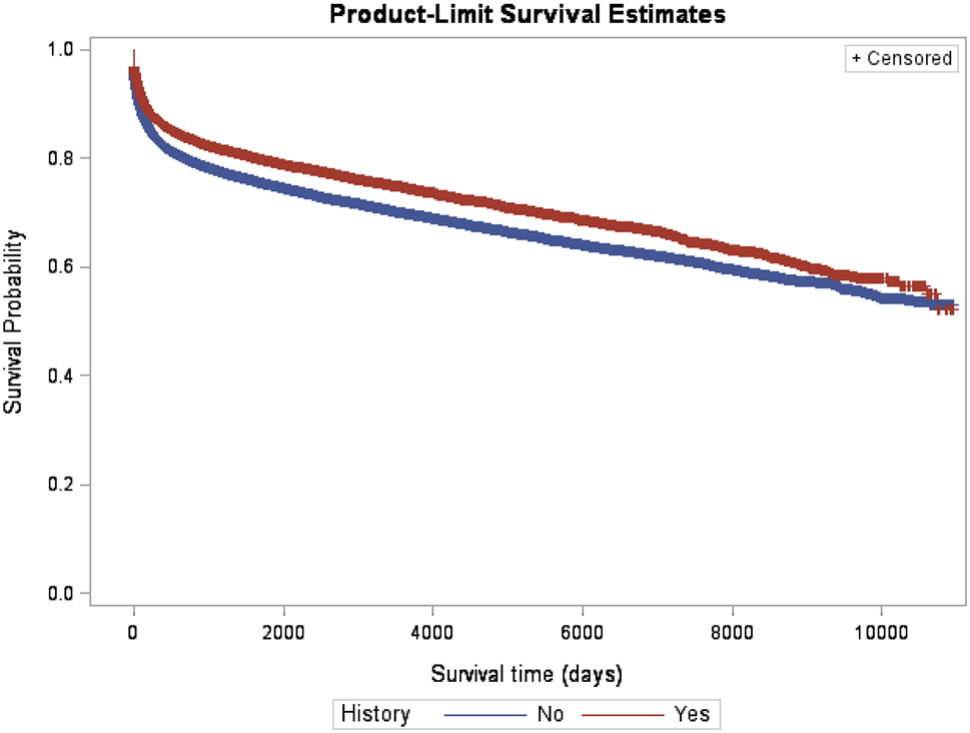
Kaplan–Meier plot by family history of venous thromboembolism (VTE). The survival was significantly higher among those with family history of VTE (p < 0.001)

From index date till the end of follow-up on 31 December 2010 (Table 2), a total of 39 066 patients with NH, 29.3% died, as compared with 25.8% patients in the YH group (Table 2). The 10 years mortality rate in patients with NH was 26.3% compared to 22.2% in YH individuals (Table 2). The survival probability is also shown in Table 2. Median follow-up time was 1 963 days for NH patients (IQR 405–4 661 days) and 2 459 days among YH patients (IQR 598–5 082 days) (p < 0.001). In Fig. 2 the Kaplan–Meier plot by family history of VTE is presented. Family history was associated with a better survival (p < 001). Table 2 indicates that the mortality rate after 10 years was lower than before 10 years. Based on survival curves in Fig. 1 we divided time to event in two time periods, time >3650 days and time ≤3650 days (Table 3). The crude estimated mortality HR for YH in time ≤3650 is 0.807 (95% CI 0.771–0.845) indicating YH have an estimated 19.3% lower mortality than NH. Estimated mortality HR for time >3650 goes up to 1.018 (95% CI 0.905–1.145). In the adjusted models adjustment was made for all variables presented in Table 1. Estimated mortality HR in adjusted model is 0.846 (95% CI 0.826–0.905) for time ≤3650 days and it is 0.995 (95% CI 0.884–1.119) for time >3650 days.

**Table 2:** Mortality rate and survival probability for patients with index date from 1981 to 2010 by family history

	No (39,066)		Yes (10,093)	
	(%)	Survival probability	(%)	Survival probability
1 week	4.9	0.952	3.9	0.962
1 month	7.1	0.929	5.5	0.945
3 months	10.8	0.892	8.4	0.916
1 year	16.9	0.828	13.3	0.864
5 years	23.2	0.751	19.2	0.794
10 years	26.3	0.698	22.2	0.745
15 years	28.0	0.654	24.1	0.699
20 years	28.8	0.614	25.2	0.652
25 years	29.2	0.572	25.7	0.593
29 years	29.3	0.531	25.8	0.521

**Table 3:** Stratified mortality hazard ratios (HRs) before and after 10 years of follow up in patients with venous thromboembolism

	Reference	Follow up time	HR^a^	95% CI		HR^b^	95% CI	
History	No	≤3650	0.807	0.771	0.845	0.864	0.826	0.905
		>3650	1.018	0.905	1.145	0.995	0.884	1.119

Crude^a^ and adjusted^b^ HRs are presented

^a^Crude model, ^b^Full model is adjusted for all variables in Table 1

### Mortality in different subtypes of VTE

Instead of adjusting for the presentation of VTE (i.e. DVT, PE, PD) we also analyzed patients with DVT, PE, and PD separately (Supplement Tables 2–4). Similar pattern were observed both in the crude model and full models with significantly decreased mortality the first 10-years of follow up for those with family history and no significant difference in mortality during follow up between 10 and 30 years. In the PD group the decreased mortality among those with family history of VTE during the first 10 years was significant only in the crude model probably due to the lower number of patients in this group (crude model 0.799, 95% CI 0.653–0.978 and full model 0.841, 95% CI 0.685–1.032). Among DVT and PE patients the decreased mortality for those with family history of VTE was significant in both the crude and adjusted models (DVT crude model 0.813, 95% CI 0.759–0.869, and DVT full model 0.921, 95% CI 0.86–0.986, PE crude model 0.807, 95% CI 0.756–0.861, and PE full model 0.826, 95% CI 0.774–0.881).

### Comorbidity related mortality

In Supplement Table 5 are shown the overall HR for comorbidities in the multivariable model. Cancer was the strongest predictor for mortality (HR = 8.11, 95% CI 7.812–8.42). Among the 10,226 NH patients who died the first 10 years after first event of VTE 56.9% had cancer compared with 53.4% of the 2225 YH patients who died (p = 0.002). After more than years of follow up only 4.9% of the 1221 NH patients who died had cancer compared with 5.6% of 376 YH patients (p = 0.604). Other comorbidity associated mortality than cancer was not significantly higher the first 10 years after VTE for NH compared with YH patients.

Other significant predictors for mortality were: congestive heart failure (HR = 1.695, 95% CI 1.584–1.815), peripheral vascular disease (HR = 1.249, 95% CI 1.158–1.346), coronary heart disease (HR = 1.171, 95% CI 1.106–1.24), cerebrovascular disease (HR = 1.495, 95% CI 1.411–1.584), diabetes mellitus (HR = 1.491, 95% CI 1.41–1.577), psychiatric disease (HR = 1.468, 95% CI 1.404–1.535), other pulmonary disease (HR = 1.504, 95% CI 1.403–1.611), and liver disease (HR = 2.197, 95% CI 1.956–2.468).

Some conditions were associated with lower mortality: pregnancy/delivery (HR = 0.222, 95% CI 0.133–0.368), hypertension (HR = 0.924, 95% CI 0.88–0.97), fracture/trauma, (HR = 0.859, 95% CI 0.791–0.933), varicose veins (HR = 0.862, 95% CI 0.779–0.953), and asthma (HR = 0.848, 95% CI 0.773–0.931).

Surgery (HR = 1.007, 95% CI 0.966–1.05) and obesity (HR = 1.002, 95% CI 0.882–1.139) were not associated with mortality.

### Other factors associated with mortality

Increasing age was associated with mortality (HR = 1.033 per year, 95% CI 1.031–1.035). Female sex (HR = 0.958, 95% CI 0.927–0.991) and high education (12 or more years compared with 9 years or less education: HR = 0.76, 95% CI 0.725–0.797) had lower mortality. Patient with both DVT and PE had slightly higher mortality compared with DVT patients (HR = 1.101, 95% CI 1.063–1.139). There was no significant difference between DVT and PE (HR = 1.035, 95% CI 0.953–1.124).

## Discussion

To the best of our knowledge this is the first paper to confirm Roosendaal’s theory in a large epidemiological study that familiarly predisposed individuals have a lower thrombotic threshold [1]. In this population-based 30-year cohort study, we found that patients with a first-time hospitalization for VTE and family history of VTE compared with those without family history of VTE had a decreased risk of dying within the first 10-years after the event. VTE patients with family history of VTE also had less provoking factors and comorbidities than those without family history of VTE. The results are somewhat surprising as an increased risk for fatal PE have been reported among patients with family history of VTE [30]. However, in the published study the reference group was the general population without family history of VTE [30]. In the present study the reference group was patients with VTE without family history of VTE. As patients with VTE have significantly much higher mortality than the general population our results are not in opposition to the published study [14]. The result of our study is in line with the idea of a thrombotic threshold presented by Rosendaal [1]. Patients with a familial predisposition for VTE needs less circumstantial factor in order to provoke a thrombotic event. This explains why several comorbidities and provoking risk factors were more common among patients without family history of VTE. Adjustment for these factors decreased the mortality HR. However, residual confounding of comorbidities is likely to exist. The mechanism behind that family history of VTE after 10 years of follow-up is not protective anymore is unclear. However, it might be related to that several comorbidities such as cancer and different cardiovascular disorders have a severe prognosis and that many of these patients have died within 10 years. There was a dramatic difference in cancer associated mortality during the first 10 years of follow up compared with after more than 10 years of follow up.

An observation worth considering is that varicose veins were linked to family history of VTE confirming a previous study showing linkage between inheritance of varicose veins and increased risk of VTE suggesting shared familial susceptibility [31]. The observation of a slightly higher prevalence of DVT among patients with family history could be related to the factor V gene paradox, as factor V Leiden is highly prevalent in Sweden [32, 33].

A limitation of the present study is that we did not include outpatients, as there is no Swedish outpatient register before 2001. Less severe thrombotic events are therefore not included, which explains why the overall 30-year mortality was not significantly different among DVT and PE patients. However, outpatient treatment in Sweden did not start in a large scale until after the introduction of Low molecular weight heparin in Sweden 1994. However, with regards to the presence or absence of family history of VTE this is most likely a non-differential bias. Strength of the study design is the high coverage and high validity of the Swedish Hospital discharge register especially for cardiovascular disorders such as VTE [23, 25, 27, 28]. Another strength is using the multi-generation register together with the Swedish Hospital Discharge Register for defining family history, which eliminates recall bias. Our finding of decreased long-term mortality risk up to 10 years after VTE is likely generalizable to many Western societies but it may not apply to all ethnic groups. A limitation is the lack of information about ethnicity. It is important to note that only Swedish born VTE cases were included in the study. As only adults with VTE between 1981 and 2010 were included this means that a large majority of patients are of Scandinavian and/or European Caucasian origin.

The observation that family history of VTE is associated with less comorbidities and provoking factors and better initial prognosis is theoretically interesting confirming Roosendaal’s theory of a thrombotic threshold [1]. However, the difference is too small to be able to use alone in the clinic but could be included in a risk assessment model.

## Conclusions

We found that patients with family history of VTE have a better initial prognosis the first 10 years after first time VTE. Patients with family history of VTE have less provoking factors and less comorbidities indicating a lower thrombotic threshold.

## Electronic supplementary material

Below is the link to the electronic supplementary material.

Supplementary material 1 (DOCX 111 KB)
