# Supplementary material for: Family history of venous thromboembolism and mortality after venous thromboembolism: a Swedish population-based cohort study
Source: J Thromb Thrombolysis. 2016 Dec 19;43(4):469–75. doi: 10.1007/s11239-016-1464-y (PMC5375967; doi:10.1007/s11239-016-1464-y)
Supplement: Supplementary file 1 — Supplementary material 1 (DOCX 111 KB) [file 11239_2016_1464_MOESM1_ESM.docx]

Supplement Table 1-5

| **Supplement Table 1. Definition of comorbidities and provoking risk factors for venous thromboembolism (VTE).** | | | | | | | |
| --- | --- | --- | --- | --- | --- | --- | --- |
| Covariates | | ICD-10 | | ICD-9 | | | ICD-8 |
| Asthma* | | J45, J46 | | 493 | | | 493 |
| Atrial fibrillation/flutter* | | I48 | | 427D | | | 427.92 |
| Cancer*** | | C00-C99 | | 140-208 | | | 140-209 |
| Cerebrovascular disease* | | I60-I69 | | 430-438 | | | 430-438 |
| Congestive heart failure* | | I50 | | 428 | | | 427.00, 427.10, |
| Coronary heart disease* | | I20-I25 | | 410-414 | | | 410-414 |
| Diabetes mellitus* | | E10-E14 | | 250 | | | 250 |
| Fractures/trauma ** | | S00-S99, T00-T14 | | 800-929, 950-959 | | | 800-929, 950-959 |
| Hypertension * | | I10-I15 | | 401-405 | | | 400-404 |
| Inflammatory bowel disease* | | K50, K51 | | 555,556 | | | 563 |
| Liver disease * | | K70-K74 | | 571 | | | 571 |
| Obesity* | | E65, E66 | | 278A, 278B | | | 277.99 |
| Peripheral vascular disease* | | I70-I79 | | 440-448 | | | 440-448 |
| Pregnancy/delivery** | | O00-O99 | | 630-676 | | | 630-678 |
| Psychiatric disease* | | F10-F99 | | 290-319 | | | 290-315 |
| Pulmonary diseases (exceptasthma)* | | J41,J42,J43,J44,J47, J60-J70 | | 491,492,494, 495, 496, 500-508 | | | 491,492,515-518 |
| Varicose veins* | | I83 | | 454 | | | 454 |
| Surgery defined by special surgery codes KVÅ 1997 (1997—2010) and operation 6 (1964-1996) | | | | | | | |
|  | KVÅ 1997 (1997—2010) | | | | Operation 6 (1964-1996) | | |
| Surgery ** | AAA-ZZZ (followed by 2 digits) | | | | 0000-8999 | | |
| *Covariates: occurring between 1968 and before or at index date for VTE; ** pregnancy/delivery, fracture/trauma, and surgery occurred within 90 days before first time VTE. *** Five years before or at index date. | | | | | | | |
|  |  | |  | |  |  | |

| **Supplement Table 2.** Stratified mortality Hazard Ratios (HRs) before and after 10 years of follow up in patients with deep venous thrombosis (DVT). Crude* and adjusted** HRs are presented. | | | | | | | | |
| --- | --- | --- | --- | --- | --- | --- | --- | --- |
|  | **Reference** | **Follow up time** | **HR*** | **95% CI** | | **HR**** | **95 % CI** | |
| **History** | **No** | <= 3 650 | 0,813 | 0,759 | 0,869 | 0,921 | 0,86 | 0,986 |
|  |  | > 3 650 | 1,069 | 0,921 | 1,241 | 1,028 | 0,886 | 1,193 |

*Crude model ** Full model is adjusted for all variables in Table 1

| **Supplement Table 3.** Stratified mortality Hazard Ratios (HRs) before and after 10 years of follow up in patients with pulmonary embolism (PE). Crude* and adjusted** HRs are presented. | | | | | | | | |
| --- | --- | --- | --- | --- | --- | --- | --- | --- |
|  | **Reference** | **Follow up time** | **HR*** | **95% CI** | | **HR**** | **95 % CI** | |
| **History** | **No** | <= 3 650 | 0,807 | 0,756 | 0,861 | 0,826 | 0,774 | 0,881 |
|  |  | > 3 650 | 0,927 | 0,757 | 1,136 | 0,912 | 0,744 | 1,118 |

*Crude model ** Full model is adjusted for all variables in Table 1

| **Supplement Table 4.** Stratified mortality Hazard Ratios (HRs) before and after 10 years of follow up in patients with combined deep venous thrombosis and pulmonary embolism (PD). Crude* and adjusted** HRs are presented. | | | | | | | | |
| --- | --- | --- | --- | --- | --- | --- | --- | --- |
|  | **Reference** | **Follow up time** | **HR*** | **95% CI** | | **HR**** | **95 % CI** | |
| **History** | **No** | <= 3 650 | 0,799 | 0,653 | 0,978 | 0,841 | 0,685 | 1,032 |
|  |  | > 3 650 | 1,125 | 0,610 | 2,077 | 1,234 | 0,665 | 2,29 |

*Crude model ** Full model is adjusted for all variables in Table 1

| **Supplement Table 5.** Adjusted mortality Hazard Ratios (HRs) for all variables included in the full model and also HR for family history before and after 10 years of follow up in patients with venous thromboembolism. | | | | | |
| --- | --- | --- | --- | --- | --- |
|  | **Reference** | **Follow up time** | **HR****** | **95 % CI** | |
| **History** | **No** | <= 3 650 | 0,864 | 0,826 | 0,905 |
|  |  | > 3 650 | 0,995 | 0,884 | 1,119 |
| **Age** |  |  | 1,033 | 1,031 | 1,035 |
| **Sex** | **Male** |  | 0,958 | 0,927 | 0,991 |
| **Education, Middle(10-11 years)** | **Low(0-9 years)** |  | 0,867 | 0,835 | 0,9 |
| **Education, Higher(12 year or more)** | **Low(0-9 years)** |  | 0,76 | 0,724 | 0,797 |
| **Education, Unknown** | **Low(0-9 years)** |  | 2,407 | 2,03 | 2,854 |
| **Group PD** | **DVT** |  | 1,101 | 1,063 | 1,139 |
| **Group PE** | **DVT** |  | 1,035 | 0,953 | 1,124 |
| **Congestive heart failure** | **No** |  | 1,695 | 1,584 | 1,815 |
| **Peripheral vascular disease** | **No** |  | 1,249 | 1,158 | 1,346 |
| **cancer** | **No** |  | 8,11 | 7,812 | 8,42 |
| **CHD** | **No** |  | 1,171 | 1,106 | 1,24 |
| **Cerebrovascular disease** | **No** |  | 1,495 | 1,411 | 1,584 |
| **Diabetes mellitus** | **No** |  | 1,491 | 1,41 | 1,577 |
| **Psychiatric disease** | **No** |  | 1,468 | 1,404 | 1,535 |
| **Other pulmonary disease** | **No** |  | 1,504 | 1,403 | 1,611 |
| **Liver disease** | **No** |  | 2,197 | 1,956 | 2,468 |
| **Pregnancy or delivery** | **No** |  | 0,222 | 0,133 | 0,368 |
| **Hypertension** | **No** |  | 0,924 | 0,88 | 0,97 |
| **Surgery** | **No** |  | 1,007 | 0,966 | 1,05 |
| **Fractures / trauma** | **No** |  | 0,859 | 0,791 | 0,933 |
| **Varicose veins** | **No** |  | 0,862 | 0,779 | 0,953 |
| **Asthma** | **No** |  | 0,848 | 0,773 | 0,931 |
| **Obesity** | **No** |  | 1,002 | 0,882 | 1,139 |
| HR=hazard ratio, CI=confidence interval, DVT=deep venous thrombosis, PD=DVT+PE, PE=pulmonary embolism , CHD=coronary heart disease | | | | | |
